# Evaluation of oocyte quality in Polycystic ovary syndrome patients undergoing ART cycles

**DOI:** 10.1186/s40738-020-00094-z

**Published:** 2021-01-05

**Authors:** Roshan Nikbakht, Razieh Mohammadjafari, Mina Rajabalipour, Mahin Taheri Moghadam

**Affiliations:** Fertility, Infertility and Perinatology Research Center, Ahvaz Jundishapur University of Medical Sciences, Ahvaz, Iran

**Keywords:** Oocyte Quality, PCOS, ART cycles, AMH

## Abstract

**Background:**

To evaluate factors affecting oocyte/embryo quality in PolyCystic Ovary Syndrome (PCOS) patients undergoing Assisted Reproductive Technology (ART) cycles.

**Methods:**

This case-control retrospective study was performed on PCOS patients referred to the infertility department of Imam Khomeini Hospital in Ahvaz from October 2017 to September 2019. Demographic and reproductive characterizations including age, gender, abortion history and infertility type (primary and secondary infertility) were extracted from patient’s records. TSH, AMH, LH, FSH, prolactin, lipid profile and blood glucose was measured. Biochemistry pregnancy was checked by determination of serum βHCG level and then, clinical pregnancy was confirmed by observing of pregnancy sac and fetal heart rate using Transvaginal USS.

**Results:**

One-hundred thirty-five patients include 45 PCOS and 90 Non-PCOS patients with mean age of 31.93 ± 5.04 and 30.8 ± 5.38 (*p* = 0.24) were considered as case and control groups respectively. Retrieved oocyte numbers were significantly higher in PCOS patients (*p* = 0.024), but there was no significant difference in number of oocyte subtypes (MI, MII and GV) between two groups. The embryo numbers and its subtypes did not differ significantly in both groups. The clinical pregnancy rate was insignificantly lower in PCOS patients (*p* = 0.066) and there was a significant correlation between retrieved oocyte numbers with age(*r*= -0.2, *p*= 0.022) and AMH level (*r* = 0.433, *p* < 0.0001) respectively. Cholesterol level had shown a positive significant correlation with number of MI oocytes (*r* = 0.421, *p* = 0.026) and MII oocytes significantly affected by age (*r*= -0.250, *p* = 0.004) and AMH level (*r* = 0.480, *p* < 0.0001). Using Receiver operation characteristic (ROC) curve analysis, the cut-off value of total number of oocytes was > 10.5 with area under curve of 0.619±0.054(sensitivity 55.56% and specificity 69.66%)

**Conclusions:**

The results of this study showed that although the number of oocytes in PCOS patients was significantly higher than non-PCOS patients, the quality of oocytes was not statistically different. The number and quality of embryos were not significantly different in both groups. Our results indicated a significant relationship between the level of AMH and the number of retrieved oocytes and embryos. We found there is a significant correlation between cholesterol level and number of MI oocytes.

## Background

PCOS is a hormonal disorder common among women of reproductive age. According to Rotterdam criteria, a woman has at least two of these three characteristics: clinical and / or biochemical hyperandrogenism, ovarian dysfunction, and Polycystic ovary Morphology (PCOM) to be diagnosed as PCOS [1]. Almost 25% of infertile couples meet the criteria for PCOS diagnosis, while the prevalence of PCOM (PolyCystic Ovarian Morphology) is estimated 33% in asymptomatic patients [2, 3]. PCOM defined as either an ovary with 12 or more follicles, ranging in size from 2^mm^ to 10^mm^, in a single plane or an ovarian volume of more than 10^mL^ without a dominant follicle [4]. This excessive number of follicles is associated with folliculogenesis abnormalities that are thought to be the result of intra-ovarian hyperandrogenism and hyperinsulinemia [5]. It should be mentioned that most of these oocytes are not mature, which leads to a decrease in pregnancy rates and an increase in abortion [6]. The oocyte quality is defined by factors such as ability to undergo meiotic maturation, fertilization, proper embryonic development and successful pregnancy [7]. These qualities are obtained thorough the follicular growth by the interaction of theca and granulose cells (GCs) [8]. Since follicular growth is disrupted in PCOS patients, especially during Controlled Ovarian Hyper stimulation (COH), a decreased number of good oocytes/embryos in ART cycles are a widespread problem. As previous studies have shown, various factors can affect the quality of the oocytes and embryo. Therefore, we decided to evaluate the quality of oocytes/embryos in PCOS patients undergoing ART cycles in the region.

## Methods

### Study Design

This case-control retrospective study was performed on PCOS patients referred to the infertility department of Imam Khomeini Hospital in Ahvaz from October 2017 to September 2019. Inclusion criteria were patients with PCOS (case group), non-PCOS patients with tubal factor or male factor (control group). Exclusion criteria included patients with Follicle Stimulating Hormone (FSH) more than 12 IU/mL, ovarian surgery history, ovarian tumor, systemic diseases, endometriosis and age more than 38 years. In this study using “Power and Sample Size” software and based on paper written by Banchhita et al. [9], in which the mean and standard deviation of the number of MII oocytes in PCOS group are 12.1 and 5.2 and in non-PCOS group are 14.3 and 4.5, respectively, the sample size for PCOS and non-PCOS groups were considered as 45 and 90 respectively. Study was confirmed by the ethical committee of Ahvaz Jundishapour University of Medical Sciences under number of IR.AJUMS.REC.1398.501 and the signed informed consent was obtained from all participants.

### Measurements

Demographic and reproductive characterizations including age, gender, abortion history and infertility type (primary and secondary infertility) were extracted from the patient’s records. Thyroid-stimulating hormone (TSH), Anti-Müllerian hormone (AMH), Luteinizing hormone (LH), FSH and prolactin hormone level were measured by enzyme-linked immunosorbent assay (ELISA) test. Biochemical factors including, Fasting Blood Sugar (FBS), Glucose Tolerance Test (GTT) (75gr), Triglyceride, cholesterol and Vitamin D level were assessed. Patients in both groups underwent ART antagonist treatment cycle. On the 3rd day of menstruation, Transvaginal USS was done. If endometrium thickness was thin or no follicles greater than 10^mm^ were seen, COH was started (Antagonist cycle). Depending on the patient’s condition, a dose of 150 or 225 units of Gonal-F (Merck, Germany) or Cinnal-F(CinnaGen, Iran) was administrated. When the follicle size reached 13 or 14^mmcetr^, Cetrorelix (Merck, Germany) 0.25 mg subcutaneous was started. Gonadotrophin and Cetrorelix continued until two follicles with 17^mm^ in size and several 15^mm^ sized follicles were seen. Oocyte retrieval was performed 34 to 36 hours after injection of 10,000 units U-HCG (Pregnyl-Chorionic Gonadotrophin) or GnRH agonist (0.5 cc Superfact-Buserline acetate) and the number and grade of oocytes were recorded which included: Metaphase II (MII), Metaphase I (MI), and Germinal Vesicle (GV). MII oocytes have excellent quality and the main goal of Controlled ovarian Hyperstimulation (COH) is to achieve maximum number of MII oocytes. MI-type oocytes are lower in maturity than MII-type and are not a favorable treatment option. GV oocytes have the lowest quality and are not a good choice for transferring. Twenty-four hours after fertilization, the embryos morphology was evaluated to check 2-pronuclear (2PN) morphology. Then if no ovarian hyperstimulation syndrome (OHSS) occurred and endometrium more than 8 mm thickness was seen, 1 or 2 embryo was transferred.

### Outcomes

Two weeks after transfer, biochemistry pregnancy was checked by determination of serum βHCG level. Then, clinical pregnancy was confirmed by observing of pregnancy sac and fetal heart rate using Transvaginal USS.

### Statistical analysis

The mean value of variables compared using independent t-student or Mann–Whitney U test and proportions were compared using the chi-square test. Correlations between the variables were determined by Pearson correlation coefficient test. All statistical analysis was performed using SPSS version 22 and P-value of less than 0.05 considered significant.

## Results

135 patients include 45 PCOS patients with mean age of 31.93 ± 5.04 and 90 Non-PCOS with mean age of 30.8 ± 5.38 (*p* = 0.24) years were considered as case and control group respectively. Although most PCOS patients had secondary infertility (31(68.9%)), primary infertility was more common in non-PCOS patients (50(55. 6%)).The mean values of infertility duration in PCOS and Non- PCOS patients were 7.4 and 6.3 years, respectively(*p* = 0.11). Although the mean level of FSH, LH and prolactin were similar, The AMH level was significantly higher in PCOS patients (*p* < 0.0001). FBS, Cholesterol and Triglyceride were non-significantly higher in PCOS patients (Table 1). The mean age of patients, smoking, alcohol consumption and addiction had not shown significant differences between two groups. Retrieved oocyte numbers were significantly higher in PCOS patients (*p* = 0.024). However, oocyte subtypes (MI, MII and GV) did not shown any significant differences between two groups.

**Table 1:** Demographic Data (PCOS & non-PCOS)

Variable		PCOS (*N* = 45)	Non-PCOS (*N* = 90)	*P*-value
**Age**		31.93 ± 5.04	30.8 ± 5.38	0.24
**Husband’s age (Year)**		35.33 ± 4.1	33.93 ± 4.3	0.074
**BMI**		25.8 ± 2.94	26.7 ± 2.8	0.069
**Alcohol**		2(4.4%)	1(1.1%)	0.25
***Addiction***		1(2.2%)	0	0.33
***Smoking***		9(20%)	14(15.6%)	0.628
**Infertility type**	Primary	14(31.1%)	50(55.6%)	0.010
	Secondary	31(68.9%)	40(44.4%)	0.010
	Infertility duration (Year)	7.4 ± 3.7	6.36 ± 3.9	0.11
**Hormonal Profile**	FSH (IU/mL)	7.78	7.122	0.69
	AMH (ng/mL)	6.67	3.74	< 0.0001
	*LH* (IU/mL)	8.38	7.04	0.47
	*Prolactin* (ng/mL)	33.34	21.56	0.082
**Metabolic Profile**	FBS (mg/dL)	98.25 ± 26.45	92.6 ± 22.7	0.26
	Cholesterol (mg/dL)	200 ± 74	158 ± 46	0.08
	TG (mg/dL)	148	133	0.63
	Vitamin D (ng/dL)	17.7	22.7	0.33

The embryo numbers and its subtypes did not differ significantly in both groups. The clinical pregnancy rate was insignificantly lower in PCOS patients (*p*=0.066) (Table 2). There was a significant correlation between retrieved oocyte numbers with age (*r*=−0.2, *p*= 0.022) and AMH level (*r*=0.433, *p*<0.0001), respectively. While the MII oocytes significantly affected by age (*r*=−0.25, *p*=0.004) and AMH level (*r*=0.480, *p*<0.0001).

**Table 2:** Oocytes, Embryos and fertilization rate comparison of PCOS and non-PCOS patients

Variable	PCOS (*N* = 45)	Non-PCOS (*N* = 90)	*P* value
**Oocyte numbers**	13.91 ± 11.54	8.96 ± 6.6	**0.024**
**MII oocyte**	9 ± 8.6	6.21 ± 5.2	0.087
***MI oocyte***	1.6 ± 3.03	1.06 ± 1.7	0.184
**GV oocyte**	2.04 ± 4	1.14 ± 1.5	0.117
***MII Ratio (MII/Total)***	0.75	0.69	0.051
**Total Embryos**	6.91	4.6	0.147
**Embryos (Grade A)**	0.11	0.11	0.759
**Embryos (Grade B)**	1.25	1.82	0.337
**Embryos (Grade C)**	3.67	2.4	0.123
**Embryos (Grade D)**	0.69	0.31	0.271
**Pregnancy rate**	13(28.9%)	41(45.6%)	0.066

**Fig. 1:**
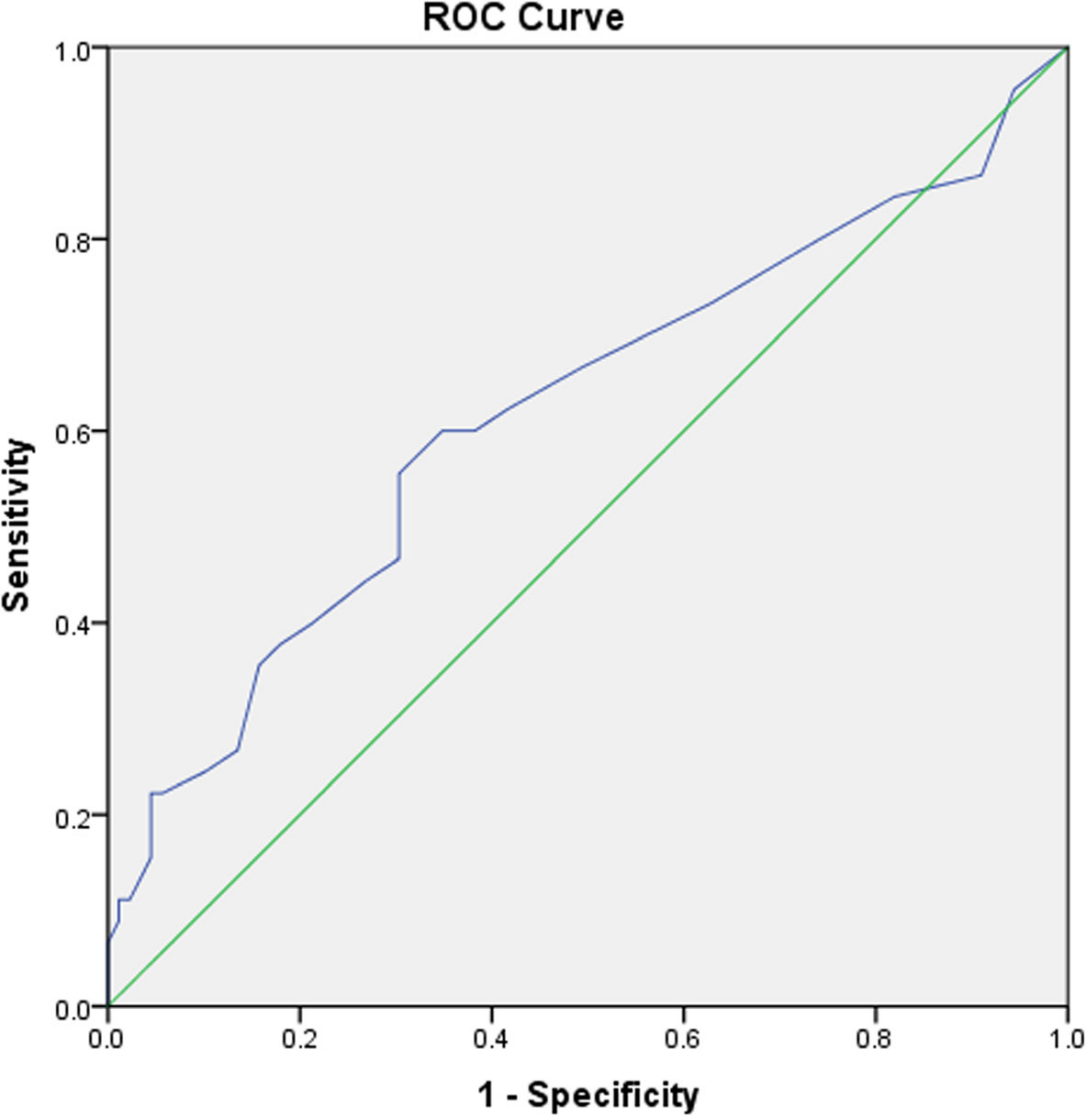
ROC curve of total number of retrieved oocyte with clinical pregnancy rate

We calculated the cut-off values of total number of retrieved oocytes, MI, MII, GV using ROC curves to predict in vitro fertilization (IVF) outcomes. Cut-off value for total oocyte number was >10.5 with area under curve of 0.619±0.054(sensitivity 55.56% and specificity 69.66%) (Fig. 1). ROC curve analysis for other parameters (MI, MII and GV) didn't result into significant cut-off value.

Moreover, Cholesterol level had shown a significant correlation with MI oocytes (*r*=0.421, *p*=0.026) (Table 3).

**Table 3:** Correlation of studied variables and oocytes characteristics

Variables	Retrieved Oocyte numbers		MII		MI		GV	
	*r*	*P value*	*r*	*P value*	*r*	*P value*	*r*	*P value*
***Age***	− 0.200^*^	0.022	− 0.250^**^	0.004	-0.111	0.203	0.022	0.799
***FSH***	-0.104	0.242	-0.099	0.262	-0.101	0.253	-0.015	0.863
***AMH***	.433^**^	0.000	.480^**^	< 0.0001	0.152	0.137	0.161	0.114
***LH***	0.073	0.419	0.045	0.620	-0.015	0.870	0.114	0.204
***TSH***	-0.006	0.943	-0.042	0.628	-0.012	0.894	-0.011	0.903
***FBS***	-0.020	0.845	-0.074	0.456	0.004	0.965	0.035	0.725
***TG***	0.004	0.982	-0.120	0.486	0.003	0.987	-0.090	0.601
***Cholesterol***	0.373	0.050	0.180	0.359	.421^*^	0.026	0.166	0.398
***Vit D***	0.007	0.943	0.066	0.503	-0.133	0.179	0.001	0.992
***Prolactin***	0.072	0.406	-0.025	0.776	-0.060	0.489	-0.025	0.770

*p-value < 0.05 **p-value < 0.01

Spearman correlation test showed negative correlation between total number of embryo and age but the total number of embryo, embryo grade C and B had positive correlation with AMH. There was no significant correlation between embryos grade A and D with the other variables (Table 4).

**Table 4:** Correlation of studied variables and embryo characteristics

Variables	Embryo numbers		A		B		C		D	
	*r*	*P value*	*r*	*P value*	*r*	*P value*	*r*	*P value*	*r*	*P value*
***Age***	− 0.197^*^	0.023	-0.147	0.091	-0.136	0.119	-0.066	0.450	-0.100	0.255
***FSH***	-0.123	0.163	-0.028	0.757	-0.046	0.606	-0.127	0.151	-0.041	0.649
***AMH***	.445^**^	< 0.0001	0.084	0.411	.374^**^	0.000	.220^*^	0.030	0.110	0.284
***LH***	0.105	0.243	-0.001	0.990	0.009	0.917	0.150	0.096	0.050	0.581
***TSH***	-0.093	0.283	-0.026	0.767	0.014	0.875	-0.125	0.150	-0.081	0.357
***FBS***	-0.092	0.354	-0.068	0.494	-0.092	0.358	0.003	0.979	-0.026	0.797
***TG***	-0.121	0.481	-0.123	0.474	-0.132	0.443	-0.006	0.971	-0.080	0.647
***Cholostrol***	0.248	0.203	-0.073	0.710	0.239	0.221	0.069	0.728	0.013	0.949
***Vit D***	0.060	0.545	0.104	0.293	0.011	0.908	0.069	0.487	0.015	0.880
***Prolactin***	-0.039	0.656	-0.014	0.873	-0.004	0.964	-0.061	0.485	0.035	0.691

*p-value < 0.05 **p-value < 0.01

## Discussion

Our results have shown the number of retrieved oocytes was significantly higher in PCOS patients rather than non-PCOS. However, we could not find any statistically significant differences in number of oocyte subtypes (MII, MI and GV), number of embryo subtypes (A, B, C and D) and pregnancy rate between two groups. It was seen that; the higher rate of retrieved oocyte numbers didn’t guarantee the higher clinical pregnancy rate in PCOS group. What is important in increasing the pregnancy rate is the quality of retrieved oocytes and embryos, not the higher number of them. Despite the higher number of retrieved oocytes, the number of high-quality oocytes and embryos didn’t differ significantly in two groups. In according to our results, Ludwig et al. [10] and Plachot et al. [11]concluded that a lower number of ‘high quality oocytes’ in PCOS compared to non-PCOS patients could be attributed to a lower fertilization rate in these cycles. In line with our findings, Fernandez et al. compared the quality of oocytes and embryos in PCOS and control groups and showed, although the number of retrieved oocytes in PCOS group was higher, the number of high-quality embryos was not significantly different between two groups [12]. Current study showed that the levels of AMH in PCOS patients were significantly higher. There was also a significant correlation between the level of AMH and the number of retrieved oocytes and embryos. In a recent study it was shown that AMH is a main predictor of OHSS in non-PCOS patients [13]. Moreover, Chen and colleagues have also shown there is a positive correlation between AMH and fertility rate, embryo quality, and oocyte number [14]. Results of ROC curve analysis showed that total number of retrieved oocytes can predict the clinical pregnancy rate significantly (cut-off = 10.5, AUC = 0.619) while there weren’t any significant cut-off values for other parameters (MI, MII and GV). In a retrospective study by Lemmen et al. [15] attempted to define the oocyte number, number of embryos transferred, children per oocyte and embryo transferred in patients under IVF/ICSI cycles. In their study the average number of oocytes needed per live born child after transfer of fresh and thawed embryo was 20. We found in our study a significant correlation between cholesterol level and number of MI oocytes. This association is being reported for the first time. Browne et al. [16] in 2009 showed that Follicular fluid HDL cholesterol is negatively correlated with embryo fragmentation and it suggests that numerous lipophilic components of HDL particles including micronutrients may be influencing the membrane and cytoplasmic properties of the oocyte with downstream effects on embryo fragmentation occurring during in vitro cytokinesis. Dyslipidemia influence the oocyte quality and fertility, and accumulating studies indicate that dyslipidemia plays a potential role in the failure to fertility through inducing oxidative stress [17]. Total cholesterol (TC) was important component of dyslipidemia. The negative associations between TC levels with number of oocytes, cleavage embryos, normal fertilized oocytes, and good quality embryos of women were consistent with previous studies [18, 19]. Some observations regarding the development of human follicle suggest governing in vitro human embryo fragmentation may depend on the cholesterol metabolism, while the processes that regulating embryo cell division rate may be independent of the cholesterol metabolism [20]. We suggest further studies at the molecular and cellular level. The current study was carried out in one of the southern regions of Iran which is geographically in a particular region in terms of diversity of infertility and one limitation of this study is that it was not a multicenter study.

## Conclusions

This study showed that the number of oocytes in PCOS patients was significantly higher than non-PCOS patients but; the number and quality of embryos were not significantly different in both groups. We also found there is a significant correlation between cholesterol level and number of MI oocytes. So, it seems that lowering blood cholesterol levels can increase the success rate of the ART cycle however, more studies are needed to confirm this finding.

## Data Availability

The datasets analyzed during the current study are not publicly available because these data are private and confidential information of infertile patients were referring to Infertility and Perinatology ward of Ahwaz Jundishapur University of Medical Sciences but are available from the corresponding author on reasonable request.

## Abbreviations

PCOS: PolyCystic ovary syndrome
PCOM: PolyCystic Ovarian Morphology
ART: Assisted Reproductive Technology
TSH: Thyroid-stimulating hormone
AMH: Anti-Müllerian hormone
LH: Luteinizing hormone

## References

[1] Escobar-Morreale HF (2018). Polycystic ovary syndrome: definition, aetiology, diagnosis and treatment. Nat Rev Endocrinol Nature Publishing Group.

[2] Murphy MK, Hall JE, Adams JM, Lee H, Welt CK. Polycystic ovarian morphology in normal women does not predict the development of polycystic ovary syndrome. J Clin Endocrinol Metab. Oxford University Press; 2006;91:3878–84. 10.1210/jc.2006-1085 16882750

[3] Sigala J, Sifer C, Dewailly D, Robin G, Bruyneel A, Ramdane N (2015). Is polycystic ovarian morphology related to a poor oocyte quality after controlled ovarian hyperstimulation for intracytoplasmic sperm injection? Results from a prospective, comparative study. Fertil Steril Elsevier.

[4] Zhu R-Y, Wong Y-C, Yong E-L (2016). Sonographic evaluation of polycystic ovaries. Best Pract Res Clin Obstet Gynaecol Elsevier.

[5] Catteau-Jonard S, Dewailly D (2009). Pathophysiology of disturbed folliculogenesis in PCOS. Médecine la Reprod.

[6] Azziz R, Carmina E, Chen Z, Dunaif A, Laven JSE, Legro RS (2016). Polycystic ovary syndrome. Nat Rev Dis Prim Nature Publishing Group.

[7] Rienzi L, Balaban B, Ebner T, Mandelbaum J (2012). The oocyte. Hum Reprod. Oxford University Press.

[8] Li Q, McKenzie LJ, Matzuk MM (2008). Revisiting oocyte–somatic cell interactions: in search of novel intrafollicular predictors and regulators of oocyte developmental competence. Mol Hum Reprod. Oxford University Press.

[9] Sahu B, Ozturk O, Ranierri M, Serhal P (2008). Comparison of oocyte quality and intracytoplasmic sperm injection outcome in women with isolated polycystic ovaries or polycystic ovarian syndrome. Arch Gynecol Obstet Springer.

[10] Ludwig M, Finas DF, Al-Hasani S, Diedrich K, Ortmann O (1999). Oocyte quality and treatment outcome in intracytoplasmic sperm injection cycles of polycystic ovarian syndrome patients. Hum Reprod. Oxford University Press.

[11] Plachot M, Belaisch-Allart J, Mayenga JM, Chouraqui A, Tesquier A, Serkine AM (2003). Qualité des ovocytes et embryons dans le syndrome des ovaires polykystiques. Gynécologie Obs Fertil Elsevier.

[12] Fernandez H. Oocyte and embryo quality in polycystic ovary syndrome. Gynécol Obstét Fertil 2003; 31: 350–354. Gynecol Obstet Fertil. 2003;31:988. 10.1016/j.gyobfe.2003.09.010 14623569

[13] Nikbakht R, Zargar M, Moramezi F, Ziafat M, Tabesh H, Sattari AR (2020). Insulin Resistance and Free Androgen as Predictors for Ovarian Hyperstimulation Syndrome in Non-PCOS Women. Horm Metab Res © Georg Thieme Verlag KG.

[14] Chen Y, Ye B, Yang X, Zheng J, Lin J, Zhao J. Predicting the outcome of different protocols of in vitro fertilization with anti-Muüllerian hormone levels in patients with polycystic ovary syndrome. J Int Med Res. SAGE Publications Sage UK: London, England; 2017;45:1138–47. 10.1177/0300060517704140 PMC5536432 28449632

[15] Lemmen JG, Rodríguez NM, Andreasen LD, Loft A, Ziebe S (2016). The total pregnancy potential per oocyte aspiration after assisted reproduction—in how many cycles are biologically competent oocytes available?. J Assist Reprod Genet Springer.

[16] Browne RW, Bloom MS, Shelly WB, Ocque AJ, Huddleston HG, Fujimoto VY (2009). Follicular fluid high density lipoprotein-associated micronutrient levels are associated with embryo fragmentation during IVF. J Assist Reprod Genet Springer.

[17] Yang X, Wu LL, Chura LR, Liang X, Lane M, Norman RJ (2012). Exposure to lipid-rich follicular fluid is associated with endoplasmic reticulum stress and impaired oocyte maturation in cumulus-oocyte complexes. Fertil Steril Elsevier.

[18] Bakeer E, Radwan R, El Mandoury A, Abd El Rahman A, Gad M, Abd El Maksoud S (2018). Anti-müllerian hormone as a diagnostic marker in Egyptian Infertile polycystic ovary syndrome females: correlations with vitamin D, total testosterone, dyslipidemia and anthropometric parameters. J Med Biochem Sciendo.

[19] Wang S, Wang J, Jiang Y, Jiang W (2020). Association between blood lipid level and embryo quality during in vitro fertilization. Medicine (Baltimore) LWW.

[20] Vaisi-Raygani A, Tavilani H, Zahrai M, Rahimi Z, Sheikh N, Aminian M (2009). Serum butyrylcholinesterase activity and phenotype associations with lipid profile in stroke patients. Clin Biochem Elsevier.

